# Hôtel Dieu

**Published:** 1833-02

**Authors:** 


### HOSPITAL REPORTS.

### HÔTEL DIEU.

*Paraplegia, arising without any obvious cause, and extending to the Superior Extremities, cured by the application of Moxas below the Cervical Region.* By M. Dance. (*Archives Gen.*)

A mason, æt. twenty-two, strong and well formed, was admitted on the 5th October; he had been indisposed for fifteen days. He had slightly wounded himself on the ring finger of the left hand, in raising a stone, and on the following day he felt himself unwell and feverish. A slight eruption of pustulous pimples then appeared on the skin, of which, however, there were no traces when he entered the hospital. At the expiration of eight days, other and more serious symptoms arose, which could not be connected with the preceding. The inferior extremities, after a sensation of numbness being felt in them, became quite paralytic, and the trunk of the body was also similarly affected. The paralysis commenced in the right inferior extremity, and then extended to the left. Both sensation and motion were destroyed, the patient not being able to feel when he was pinched very hard. The limbs were free from pain, stiffness, or convulsive movements; and, when they were raised, they fell like lifeless masses. The same loss of sensibility existed over the whole surface of the abdomen. The bladder was distended with urine, and it was necessary to draw it off with the catheter. No evacuation from the bowels for six days. Respiration was carried on principally by the action of the diaphragm, and consequently there was a marked elevation of the abdomen at each inspiration, while the ribs remained motionless. The patient could not cough. His speech was not affected, and his intellect remained clear. The upper extremities soon became paralytic; the right being weaker than the left; neither could be completely raised from the side. The pulse was small, weak, and rather slow. Skin cold and mottled; expression of countenance natural; appetite moderate; nights disturbed.

For such symptoms no cause could be assigned from the information derived from the patient. He had had no fall; had suffered from no contusion nor violent efforts; nor had he been exposed to severe cold, which might have affected the spine. There was no pain along the vertebral column, either previous or subsequent to the symptoms of paralysis; neither motions of the body, percussion upon the spinous processes of the vertebræ, nor the application of a sponge soaked in hot water along the spine, produced any pain.—Sulphur baths were ordered; two grains of calomel; purgative clyster; broth diet. This treatment was continued for several days without advantage; the paralytic affection remained as before, and still without stiffness or pain. Respiration became very laborious, for want of the assistance of the pectoral muscles, and sometimes the patient complained of a sense of suffocation. Pulse continued feeble, small, and slow; skin cold; involuntary flow of urine; bowels never open without enemas. Various parts of the body, upon which he lay, were now very painful from excoriations, and even partial sloughing.

On the 15th October two moxas were applied on the back of the neck at the lower part. Sulphur baths continued.

17th. There were some feeble signs of sensibility in the lower limbs, particularly the left; state of the pulse the same, and still incontinence of urine.

23d. Return of sensibility more decided, but no power of motion restored.

November 6th. Slight motions were perceived in the muscles of the inferior extremities, but still not sufficient to move the limbs; sensibility of all the parts now restored to its natural degree; urine only flows involuntarily during the night; and the elevation of the thorax proves that the muscles of that region begin to assist in the act of respiration. The wounds from the moxas were kept open by several peas.

14th. Still improving. The patient can now raise himself, and bend and extend his legs as if he were in perfect health, but he cannot get out of bed and stand upright. Excoriations beginning to heal.

Towards the end of the month the improvement was still greater, and on the 6th December the patient could walk about the ward with a stick. The 28th, he quitted the hospital perfectly cured. He said he felt able to walk several miles. He was advised to keep up the discharge from the moxas for some time.

The seat of the disease in the above case cannot be doubted, if we judge from the symptoms: they clearly indicated a deep-seated affection of the spinal marrow, for, excepting the diaphragm, all the organs under the influence of the spinal cord were completely paralysed. Even the heart seemed to be thus affected, from the weakness and slowness of its pulsations; and it may be observed, that this circumstance tends to confirm the results of the ingenious experiments of Legallois upon the spinal marrow, which were made for the purpose of demonstrating its influence upon the motions of the heart. But, although the seat of the disease may be strongly presumed, much doubt remains as to its precise nature. Had there been inflammation of the membranes or of the substance of the spinal marrow, or was there effusion in the spinal canal? The complete absence of pain in the direction of the spine; the total absence of rigidity, or of contraction or spasmodic movements in the paralysed parts; the absence of fever, and other signs of sympathetic irritation, appear to prove that there was compression upon the part rather than inflammation of it. But, whatever was the nature of the disease, it appears evident that, but for the assistance of art, it would have terminated fatally; and the principal benefit was certainly owing to the use of the moxas, the employment of which, in such cases, has frequently been found of service.

